# Fire Safety of Polymer Nanocomposites: An In-Depth Analysis Based on Functional Mechanisms

**DOI:** 10.3390/ma19122558

**Published:** 2026-06-12

**Authors:** Junfan Liu, Kangping Li, Guangyi Zhang, Bihe Yuan

**Affiliations:** School of Safety Science and Emergency Management, Wuhan University of Technology, Wuhan 430070, China

**Keywords:** flame-retardant, fire safety, polymer nanocomposites, nanofillers

## Abstract

Polymeric materials face serious fire-safety challenges in construction, electrical and electronic devices, and aerospace because of their high heat release, melt-dripping tendency, and severe smoke and toxic emissions during burning. This review systematically summarizes the roles of nanofillers in the fire safety of polymer nanocomposites across three interconnected levels: functional mechanisms, regulatory factors, and macroscopic fire behavior. It focuses on four main mechanisms, namely physical barriers, catalytic charring, free-radical scavenging, and rheological network reconstruction, and further discusses how filler geometry, loading level, interfacial compatibility, dispersion state, and spatial orientation regulate fire-safety performance. By linking these factors to time to ignition, thermal stability, heat release, flame spread, and smoke emission and toxicity, the review clarifies the intrinsic structure–mechanism–property relationships. Current studies indicate that the fire-safety improvements provided by nanofillers do not arise from any single effect, but from their coupled regulation of heat transfer, mass transfer, radical reactions, and high-temperature rheology throughout thermal degradation, ignition, heat release, flame spread, and smoke and toxic-gas emission. Remaining challenges include the lack of unified evaluation criteria, limited in situ mechanistic evidence, and insufficient application-oriented rational design. Future work should establish verifiable, comparable, and predictive structure–mechanism–property relationships for polymer nanocomposites.

## 1. Introduction

**Figure 1:**
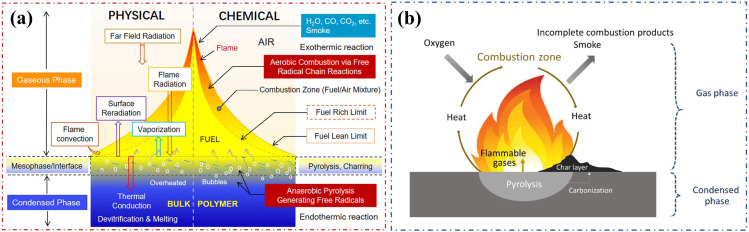
(**a**) Typical zones and physical/chemical processes during polymer combustion. Reproduced with permission from ref. [11]. (**b**) Typical combustion cycle of polymers. Reproduced with permission from ref. [5].

Polymers, such as polyethylene (PE), polypropylene (PP), polyurethane (PU), and epoxy resin (EP), have been widely used in aerospace, construction engineering, and electrical and electronic applications because of their low weight, high specific strength, and good processability. However, their hydrocarbon-based chemical structures make them inherently flammable and thus associated with significant fire hazards [1,2,3]. Owing to their high heat of combustion, polymeric materials release large amounts of energy during burning and establish a self-sustaining heat feedback loop [4,5]. In real fire scenarios, their extremely high peak heat release rate often causes a rapid rise in ambient temperature and can readily induce flashover [6,7]. Meanwhile, the rheological failure of thermoplastic polymers at elevated temperatures leads to a sharp decrease in melt viscosity. The resulting melt dripping can not only trigger secondary fires, but also form flowing fires or pool fires, thereby causing more severe fire consequences [6,8]. Figure 1 schematically illustrates the phase-zone distribution and the associated heat and mass transfer during polymer combustion. In addition, the smoke generated in polymer fires contains both toxic gases and soot particles, which can rapidly cause asphyxiation, poisoning, and loss of visibility, and are the main factors responsible for fire-related casualties [9,10]. Given the widespread use of polymeric materials in modern infrastructure and their high fuel load, the development of highly efficient flame-retardant technologies has become essential for reconciling material multifunctionality with public safety.

Traditional flame retardants, especially halogen- and phosphorus-based systems, have improved the fire safety of polymers to some extent. However, the trade-offs among flame-retardant efficiency, material performance, and environmental safety have become increasingly evident. During combustion, halogenated flame retardants, including chlorinated and brominated compounds, may release toxic or corrosive species such as hydrogen chloride, hydrogen bromide, and dioxin-like by-products, which not only pollute the environment but also pose risks to human health [12,13]. In particular, several brominated flame retardants have raised persistent environmental and toxicological concerns, and some of them have been restricted, phased out, or withdrawn from specific polymer applications under increasingly stringent regulations. By contrast, phosphorus-based flame retardants, as halogen-free alternatives, generally show better environmental compatibility. Nevertheless, their high loading requirements often impair the mechanical properties of polymers, such as tensile strength and impact toughness, and their volatility may in some cases increase the toxicity of fire effluents [14,15]. Similarly, inorganic flame retardants such as aluminum trihydroxide (ATH) have lower toxicity and better environmental performance, but their high loading levels not only increase material weight but also adversely affect processability and mechanical properties [3,16]. Therefore, under increasingly stringent environmental regulations, the development of low-toxicity, high-efficiency, and sustainable halogen-free flame-retardant materials has become an important direction in materials science research. Inherently flame-retardant polymers also provide an important route to polymer fire safety because their thermally stable aromatic or heteroatom-containing backbones can enhance char formation and reduce flammability. However, their practical use is often constrained by cost, processability, and application-specific performance requirements. In this context, nanotechnology-based flame-retardant modification has attracted increasing attention as a complementary strategy, especially for commodity polymers and polymer systems requiring balanced fire safety, processability, and overall material performance.

Nanomaterials can significantly enhance the flame retardancy of polymers at low loadings because of their high specific surface area and favorable interfacial interactions, thereby reducing the adverse effects that conventional high filler contents often have on mechanical properties [12,17]. They can also improve fire safety through mechanisms such as catalytic charring and free-radical scavenging, which promote the formation of a stable char layer on the polymer surface and interfere with combustion chain reactions. Graphene [18,19], carbon nanotubes (CNTs) [20,21], and black phosphorus (BP) [22], for example, have been shown to improve thermal stability while reducing heat release and smoke production. On this basis, emerging nanomaterials such as metal–organic frameworks (MOFs), MXenes, and layered silicates are being increasingly explored for flame-retardant polymers because of their low toxicity, functional diversity, and potential for sustainable design [23].

Despite the progress achieved in polymer nano-flame-retardant systems, a systematic understanding of how nanofillers act at different combustion stages, and how these effects are translated into macroscopic improvements in fire safety, is still limited. Existing studies have provided extensive results on thermal stability enhancement, heat release reduction, and smoke toxicity suppression, but the functional boundaries of nanofillers in different systems, their synergistic conditions, and their spatiotemporal evolution during combustion still require further clarification. In this review, we focus on the fire safety of polymer nanocomposites and examine the roles of nanofillers in ignition, heat release, flame spread, and smoke toxicity from the perspectives of physical barrier effects, catalytic charring, free-radical scavenging, and rheological network reconstruction. We further discuss how filler geometry, interfacial characteristics, dispersion state, and spatial orientation influence flame-retardant behavior, with the aim of providing a basis for the rational design and fire safety optimization of polymer nanocomposites.

## 2. Flame-Retardant Functional Mechanisms of Nanomaterials

Polymer combustion is a complex multiphase process involving heat transfer, mass transfer, and free-radical chain reactions. Owing to their unique topological structures, high specific surface areas, and active surface physicochemical sites, nanomaterials can intervene in the thermal degradation and combustion of polymers and alter the associated kinetics. Understanding these microscopic processes is important for clarifying the structure-property relationships of flame-retardant nanocomposites and for guiding the rational design of advanced fire-safe materials. In this section, the flame-retardant functions of nanofillers are discussed from four aspects: physical barrier effects and catalytic charring in the condensed phase, free-radical scavenging in the gas phase, and rheological network reconstruction at elevated temperatures.

### 2.1. Physical Barrier Effect

**Figure 2:**
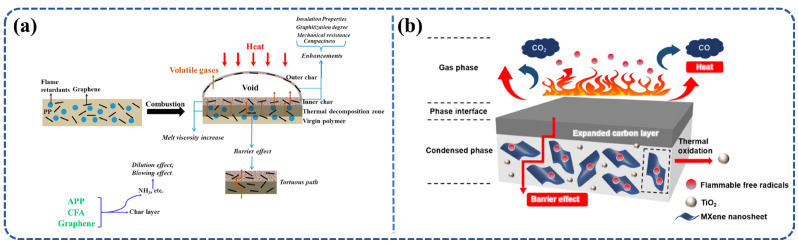
(**a**) Flame-retardant functional mechanism of graphene in the IFR/PP system. Reproduced with permission from ref. [35]. (**b**) Flame-retardant mechanism of MXene. Reproduced with permission from ref. [25].

The physical barrier effect is one of the main flame-retardant mechanisms of two-dimensional (2D) nanosheets and high-aspect-ratio nanofillers, such as graphene [18,19], MXenes [24,25], layered double hydroxides (LDHs) [26,27], and molybdenum disulfide (MoS_2_) [28,29]. Figure 2 presents two representative barrier-based flame-retardant mechanisms of 2D nanofillers. Because of their high specific surface areas and anisotropic geometries, these nanofillers can create a tortuous diffusion path in the polymer matrix [30,31]. During combustion, this tortuous path hinders both heat and mass transfer. On the one hand, it extends the diffusion path and escape time of pyrolysis volatiles to the material surface, thereby reducing the fuel supply to the gas-phase flame zone. On the other hand, it delays the penetration of external oxygen into the material interior and weakens heat transfer from the flame to the underlying unburned matrix [32]. In this way, the physical barrier effect reduces the efficiency of heat and mass exchange and weakens the self-sustaining nature of combustion.

During actual combustion, the formation of a physical barrier is also accompanied by the dynamic evolution of nanofillers in the condensed phase. In some melt-processable systems, as the polymer is heated and melts, and its viscosity decreases, nanofillers dispersed in the matrix can gradually migrate and accumulate at the material surface under the combined effects of melt flow and the evolution of degradation gases [33,34]. These surface-enriched fillers can further combine with the residual char of the matrix, which favors the formation of a continuous protective layer on the material surface and further inhibits the transfer of heat, oxygen, and combustible volatiles. However, surface enrichment does not necessarily lead to complete coverage. The continuity of the protective layer is still affected by factors such as bubbling intensity, melt flow, and the char-forming behavior of the polymer matrix.

### 2.2. Catalytic Charring Effect

The catalytic charring effect reflects a shift in flame-retardant mechanisms from passive physical barrier effects to active chemical regulation. Unlike mechanisms that mainly rely on delaying the diffusion of volatile products, nanomaterials with specific active sites can alter the thermal degradation pathways of the polymer matrix in the condensed phase [36,37]. During the initial stage of heating, these catalytically active centers can induce and accelerate the dehydrogenation, aromatization, and cross-linking of polymer chains, thereby prematurely converting hydrocarbon segments that would otherwise decompose into flammable low-molecular-weight volatiles into highly cross-linked solid char [38,39]. Catalytic charring not only increases the final char yield, but also improves the mechanical and thermal stability of the char layer by increasing its degree of graphitization [40]. Recent studies indicate that the catalytic charring efficiency of nanofillers in the condensed phase depends strongly on the nature of their surface active sites. The most widely studied mechanisms mainly involve two pathways: catalysis by surface solid acid sites and catalysis by multivalent transition metal ions.

Solid acid catalysis acts primarily by changing the kinetic selectivity of polymer degradation [37]. Nanomaterials containing Lewis or Brønsted acid sites, such as phosphorus-containing fillers capable of generating polyphosphoric acid upon heating, can participate in the early-stage degradation process when their acid-release temperature window is well matched with the initial degradation temperature of the polymer matrix [13]. This in situ acid catalysis lowers the activation energy of reactions such as dehydration, cyclization, and cross-linking, and thus directs the carbon skeleton, which would otherwise undergo scission to form small gaseous fuels, toward the formation of cross-linked char precursors [14,41,42]. In essence, the formation of such an acid-catalyzed cross-linked network increases carbon retention in the condensed phase and correspondingly reduces the release of combustible gases to some extent. To make the role of solid-acid-related catalytic sites clearer, representative systems involved in acid-catalyzed charring are summarized in Table 1.

**Figure 3:**
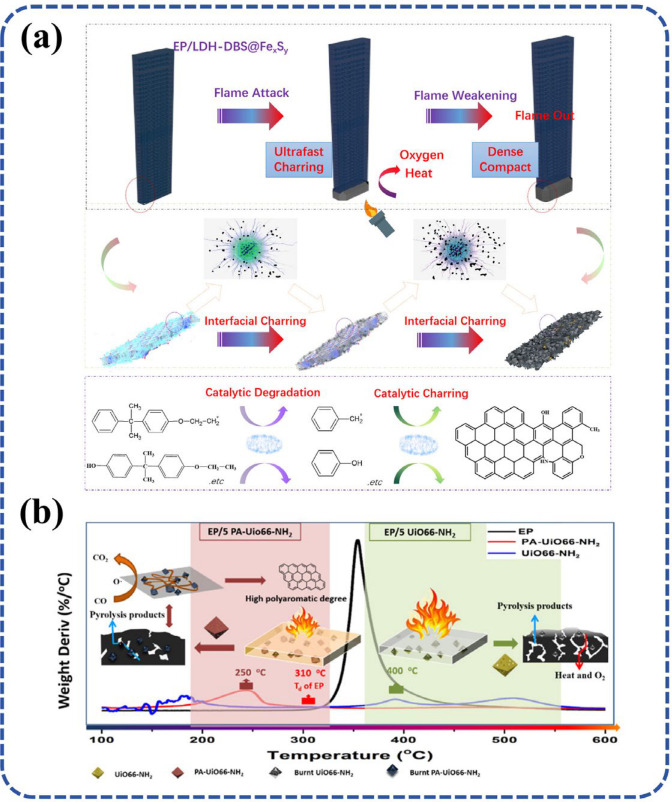
(**a**) Catalysis-tailored ultrafast interfacial charring mechanism of EP/LDH-DBS@FexSy at the macro-, meso-, and microscale. Reproduced with permission from ref. [50]. (**b**) Regulation of EP pyrolysis pathways and char-layer structures by PA-UiO66-NH_2_. Reproduced with permission from ref. [36].

By contrast, transition metal catalysis is particularly important for improving the high-temperature stability, graphitization degree, and compactness of the residual char. Transition-metal-containing nanofillers, such as MOF derivatives and two-dimensional transition metal carbides/nitrides (MXenes) containing Ni, Co, Fe, or Ti, can provide active sites with both redox and Lewis acid characteristics during combustion [43,44,45]. These active sites promote the dehydrogenation, cross-linking, and catalytic charring of polymer pyrolysis fragments, thereby favoring the formation of a continuous and compact char layer. In the intermediate and later stages of combustion, these active sites can further promote the aromatization and ordered rearrangement of amorphous char, gradually transforming it into graphitized char with higher thermal stability and better mechanical integrity [46,47]. The resulting char layer is usually smoother, denser, and less prone to cracking, which allows it to better resist internal bubble expansion and external flame-induced flow disturbance, thereby continuously hindering the transfer of heat, oxygen, and combustible volatiles [48,49]. Figure 3 compares two representative catalytic-charring pathways in epoxy systems, corresponding to transition-metal-catalyzed ultrafast interfacial charring and acid-functionalized MOF-regulated pyrolysis/charring behavior.

### 2.3. Free-Radical Scavenging and Chain-Reaction Intervention

**Figure 4:**
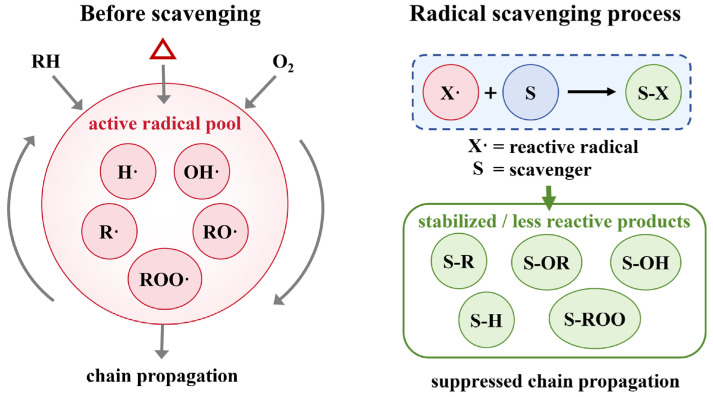
Schematic illustration of radical scavenging and suppression of radical chain propagation during combustion.

The sustained combustion of polymers depends on chain reactions involving highly reactive radicals such as H· and OH· in the flame zone. Therefore, the introduction of active components that reduce the concentrations of these key radicals and interfere with chain-transfer and branching processes provides an important chemical route for weakening flame self-sustainment [3,51]. Unlike conventional 2D inorganic nanofillers, which mainly act through condensed-phase physical barriers or catalytic charring, some nanosystems with specific electronic structures or volatile active groups can directly intervene in the radical reaction network. Depending on where the reaction occurs and which radicals are targeted, this mechanism can be divided into macromolecular radical trapping in the condensed phase and small-radical quenching in the gas phase [38,52]. Figure 4 schematically illustrates how radical scavengers reduce the concentration of reactive radicals and suppress radical chain propagation during combustion.

In the condensed phase, fullerene (C_60_) and its derivatives are representative systems. Because of its highly conjugated π-electron network, C_60_ shows high addition reactivity toward free radicals and can act as an efficient “radical sponge” in polymer melts [11]. During the initial stage of thermo-oxidative degradation, C_60_ can capture macromolecular radicals, such as alkyl and alkoxy radicals, generated by polymer backbone scission. This coupling process, which occurs in the condensed phase or at the gas–solid interface, retards the continuous cleavage of polymer chains and thus suppresses the formation of combustible low-molecular-weight volatiles at the source [53].

By contrast, gas-phase radical quenching depends on whether the nanosystem can release active fragments at elevated temperatures. BP and phosphorus-functionalized nanosystems are typical examples in this respect [22]. During high-temperature pyrolysis, these systems can release phosphorus-containing species such as PO·, HPO·, and PO_2_· into the gas phase [54,55]. After entering the flame front, these fragments rapidly undergo recombination and quenching reactions with active H· and OH· radicals, thereby interrupting gas-phase chain reactions [56,57]. In addition, some molecularly engineered hybrid systems can provide coupled intervention in both the gas and condensed phases. In 9,10-dihydro-9-oxa-10-phosphaphenanthrene-10-oxide (DOPO)-functionalized C_60_ composites, for example, phosphorus-containing fragments derived from DOPO dissociate at elevated temperatures and enter the gas phase to quench small-radical chain reactions, whereas the C_60_ carbon framework remains in the condensed phase and continues to trap macromolecular radicals [58].

### 2.4. Viscoelastic Network and Anti-Dripping

Melt dripping of thermoplastic polymers at elevated temperatures is a critical behavior governing flame spread because it substantially alters the heat and mass transfer characteristics within the combustion zone. Its impact on fire safety is strongly scenario-dependent. On the one hand, flaming drips can readily ignite surrounding combustibles, thereby causing secondary fires or pool fires [59]. On the other hand, in specific test scenarios such as the Underwriters Laboratories 94 (UL-94) vertical burning test, dripping can remove part of the polymer fuel and heat from the pyrolysis zone, which may facilitate the self-extinction of the residual material [60]. Therefore, complete elimination of dripping is not a universal objective for all nano-flame-retardant systems. Rather, the physical contribution of nanofillers lies mainly in regulating dripping behavior by altering the high-temperature rheology of the polymer melt [6].

**Figure 5:**
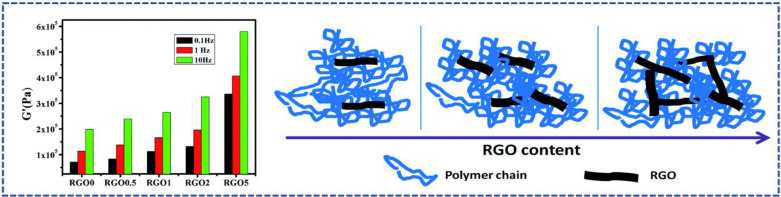
Schematic of the storage modulus enhancement induced by increasing RGO loading and the corresponding network formation process within the polymer matrix. Reproduced with permission from ref. [65].

The fundamental mechanism underlying this rheological regulation is the formation of a physical percolation network in the polymer matrix through the introduction of high-aspect-ratio or two-dimensional nanofillers. Kashiwagi et al. first elucidated this process through high-temperature rheological studies of polymer systems containing CNTs [20,21,61]. Their results showed that when CNTs reach an extremely low rheological percolation threshold, approximately 0.5–1.0 wt%, the nanocomposite melt exhibits a pronounced solid-like response under low-frequency shear, as indicated by a distinct low-frequency plateau in the storage modulus (G′) [62]. Subsequent studies on LDHs [63], montmorillonite (MMT) [64], and graphene oxide (GO) [65,66] further showed that high-aspect-ratio or layered nanofillers can restrict polymer chain mobility by forming constrained rheological structures (Figure 5). As a result, the composite melt exhibits higher viscosity and a stronger solid-like response under low-shear conditions, thereby reshaping its flow and dripping behavior [67,68].

This rheology-controlled melt viscoelasticity has a decisive but complex influence on the macroscopic dripping and combustion behavior of materials in real fire scenarios [59,69]. In cone calorimeter tests, the high melt viscosity and solid-like response induced by the nanonetwork help suppress vigorous bubbling, retard the escape of volatile degradation products, and promote the stable formation of an intumescent surface char layer, thereby reducing the heat release rate [70]. However, as noted by Kiliaris et al. [46] and Seah et al. [71], excessively high melt viscosity during UL-94 testing can retain the polymer melt in place and maintain continuous flame attack on the matrix. This reduces the ability of the material to self-extinguish through dripping-assisted heat removal and may lead to a lower flame-retardant rating. In essence, the viscoelastic network and anti-dripping mechanisms arise from the ability of nanofillers to reshape melt stability through rheological confinement. Their fire-safety benefits therefore need to be evaluated in relation to specific thermodynamic conditions and test scenarios.

## 3. Key Regulatory Factors of “Structure-Mechanism-Property” Relationships

The flame-retardant performance of nanofillers is closely related to their structural state within the polymer matrix. Even for the same filler type, differences in loading level, dispersion state, interfacial characteristics, and spatial arrangement can markedly alter the way in which a given mechanism operates and, consequently, the final fire performance. The same mechanism may therefore be enhanced, weakened, or even counteracted in different systems. Building on the above mechanistic analysis, it is necessary to further examine the key structural factors that govern the activation and effectiveness of these mechanisms. This section focuses on the effects of nanofiller geometry and loading level, interfacial optimization and dispersion, spatial orientation, and multi-mechanism coupling and hybrid design on fire safety performance.

### 3.1. Geometric Dimensions and Loading of Nanofillers

The geometric dimensions and loading of nanofillers jointly determine how efficiently they function in the polymer matrix. In general, improvements in flame retardancy do not scale linearly with filler loading [72]. At very low loadings, fillers are mostly present as isolated entities. Only when the concentration reaches a critical regime do the physical connectivity of the system and the extent of effective interfacial contact change substantially [4,73,74]. This effective regime depends strongly on filler geometry. For example, in some well-dispersed one-dimensional (1D) CNT-based systems, rheological features associated with the formation of a continuous network can already be detected at loadings of approximately 0.5–1.0 wt% [21,75]. By contrast, 2D nanosheet systems such as LDHs or MMT generally require higher loadings to evolve from isolated sheets into structures with greater coverage and stronger connectivity [31,63,74]. Therefore, filler dimensions and loading levels are fundamental structural variables that determine whether microscopic flame-retardant mechanisms can be effectively triggered.

In the physical barrier mechanism, filler geometry strongly influences the efficiency with which internal barrier structures are formed. Once the loading of 2D nanosheets such as graphene or MXenes reaches the effective regime, the sheets are more likely to overlap and interconnect, forming a more continuous and tortuous diffusion pathway in the condensed phase and thereby delaying the escape of underlying combustible volatiles [30,76,77]. In catalytic charring systems, filler loading directly affects the density of active sites in the condensed phase [78]. In nanosystems containing transition metals or MOFs, low loading levels provide too few active sites to substantially affect the thermal degradation pathways of macromolecules [39]. Only when the loading reaches the effective regime can catalytic dehydration and cross-linking reactions make a substantial contribution to the compactness of the residual char [79,80]. Similarly, for chemical intervention systems that rely on free-radical scavenging, such as fullerenes or BP, the filler loading determines the number of active components available for reaction and thus affects their overall contribution to flame retardancy [22,81]. In addition, in the regulation of high-temperature rheological behavior, filler dimensions and loading levels jointly influence the emergence of the rheological percolation threshold.

However, increasing nanofiller loading does not lead to monotonic improvements in flame-retardant performance. For 2D nanosheets, high loading levels tend to promote aggregation and restacking, which reduce the effective specific surface area, lead to uneven sheet coverage, and disrupt continuous barrier pathways [77,82]. For zero-dimensional nanoparticles or 1D nanotubes, excessive loading can cause particle agglomeration or nanotube entanglement, thereby reducing effective interfacial contact and active-site exposure and weakening the uniformity and continuity of the residual char layer [79,83]. In addition, excessive filler loading increases processing viscosity and deteriorates mechanical properties, which runs counter to the basic requirement of low loading and high performance in nano-flame-retardant systems [30,46]. Therefore, filler geometry determines the minimum loading required to trigger effective flame-retardant mechanisms, whereas an appropriate loading level is essential for avoiding severe agglomeration, preserving engineering properties, and ensuring that the nanostructure can stably exert its physical and chemical effects.

### 3.2. Interfacial Optimization and Dispersion of Nanofillers

Interfacial compatibility and dispersion state directly affect the effective interfacial area of nanofillers and the accessibility of their active sites within the polymer matrix [17,84]. Because of their high surface energy and interparticle interactions, nanofillers are prone to aggregation in polymer systems [44,85]. When filler–matrix interfacial compatibility is insufficient, particle agglomeration or the restacking of 2D nanosheets can readily occur [74,79,86]. Such dispersion defects reduce the accessible specific surface area of the fillers and the uniformity of their spatial distribution, thereby weakening the formation of continuous barrier pathways and limiting the exposure and accessibility of chemically active sites [12,23]. In poly(methyl methacrylate) (PMMA)/single-walled carbon nanotube (SWNT) systems, well-dispersed nanotubes can form a continuous network-like surface layer during combustion, whereas poor dispersion more readily leads to a discrete island-like residual structure accompanied by higher heat release [20]. This result indicates that dispersion quality alone can significantly alter the structural evolution of the condensed phase and the resulting flame-retardant effect.

**Figure 6:**
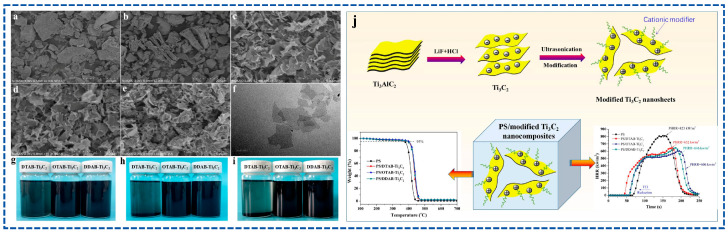
Morphology and dispersion of functionalized Ti_3_C_2_ MXene nanosheets: (**a**–**e**) SEM images of Ti_3_AlC_2_, Ti_3_C_2_, DTAB-Ti_3_C_2_, OTAB-Ti_3_C_2_, and DDAB-Ti_3_C_2_; (**f**) TEM image of Ti_3_C_2_ nanosheets; (**g**–**i**) dispersion states of modified Ti_3_C_2_ nanosheets in DMF; (**j**) schematic illustration of the preparation and flame-retardant effect of PS/modified Ti_3_C_2_ nanocomposites. Reproduced with permission from ref. [90].

Organic modification or surface treatment is commonly used in 2D systems such as layered silicates and LDHs to improve compatibility with the organic matrix and to increase the degree of intercalation and exfoliation [87]. In layered silicates, the transformation of the filler surface from hydrophilic to organophilic is a prerequisite for polymer chains to intercalate into the interlayer galleries and achieve a higher degree of exfoliation [74]. Improved dispersion and exfoliation increase the spatial coverage of nanosheets at a given loading level, thereby facilitating the construction of tortuous diffusion pathways in the condensed phase [88]. Emerging 2D fillers such as MXenes follow the same general principle: surface modification and composite design reduce restacking and strengthen interfacial interactions, thereby improving the effective utilization of the layered structure [89]. A representative example is provided by cationically modified Ti_3_C_2_ nanosheets in polystyrene (PS). Surface functionalization enlarges the interlayer distance, suppresses nanosheet restacking, and improves dispersion stability, as reflected by enhanced thermal stability and reduced heat release (Figure 6) [90].

Dispersion state also affects the accessibility of active sites in chemically active flame-retardant systems [91]. For fillers that rely on catalytic charring or free-radical scavenging, improved dispersion increases the spatial exposure of active sites in the matrix and thereby enhances their interaction with macromolecular pyrolysis intermediates or active free radicals [11,92]. In DOPO-functionalized C_60_ systems, the good solubility of DOPO groups in organic solvents facilitates the dispersion of C_60_ in polyolefins [58]. At the same time, this system combines the condensed-phase radical trapping of C_60_ with the gas-phase free-radical quenching effect of DOPO. In such multiphase intervention systems, the direct significance of improving dispersion lies in increasing the spatial exposure and accessibility of both intrinsic and newly introduced active sites.

### 3.3. Spatial Orientation of Nanofillers

**Figure 7:**
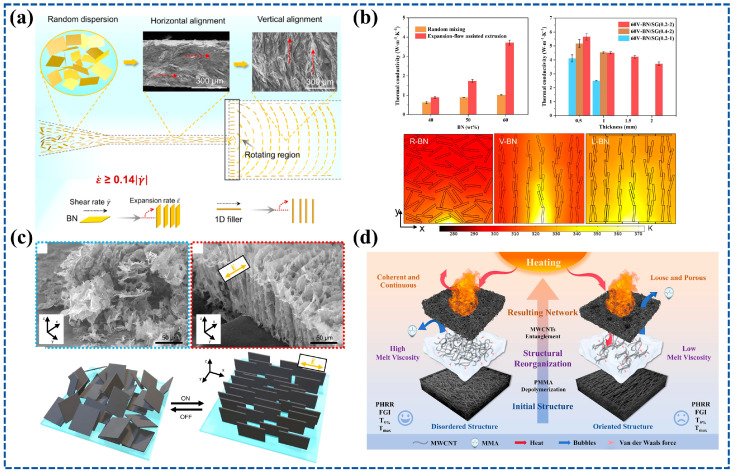
(**a**,**b**) Expansion-flow-assisted alignment of BN platelets and its effect on thermal conductivity in polymer composites. Reproduced with permission from ref. [95]. (**c**) Electric-field-induced alignment of MXene. Reproduced with permission from ref. [100]. (**d**) Contrasting combustion evolution of disordered and oriented PMMA/MWCNT networks. Reproduced with permission from ref. [103].

The spatial orientation of anisotropic nanofillers in polymer matrices, such as 1D nanotubes and 2D nanosheets, is generally determined by either passive rearrangement under processing flow fields or active alignment induced by external fields [93,94]. During melt processing operations such as extrusion and injection molding, shear and extensional flow fields drive fillers to deflect and align along the flow direction [95]. Active strategies such as electric fields, magnetic fields, mechanical stretching, and directional freeze-casting can also overcome the tendency toward random distribution or agglomeration and thereby construct specifically ordered networks [93,96]. Figure 7a,b illustrate a representative flow-field-assisted route for regulating the orientation of anisotropic fillers and its effect on thermal conductivity, whereas Figure 7c presents the reversible electric-field-induced reorientation of Ti_3_C_2_T_x_ MXene sheets. In addition, because of the shear-rate gradient and wall-friction effects inside processing molds, the near-surface region usually experiences high shear and thus strong filler alignment, whereas the core retains a more random distribution under lower shear. As a result, a skin–core structure is often formed, with an ordered skin and a more disordered core [97].

Spatial orientation directly changes both the geometric relationship between fillers and the direction of heat and mass transfer and the connectivity of the filler network [73,88]. For 2D layered fillers, when their basal planes are aligned parallel to the material surface and thus perpendicular to the gas-diffusion direction, the orientational order parameter approaches the ideal state. This highly ordered arrangement maximizes tortuosity in the condensed phase and thereby strengthens the labyrinth effect that physically blocks combustible volatiles and oxygen [88,98]. When the fillers are oriented perpendicular to the material surface, directional pathways for through-thickness heat or charge transport can be established more readily [99,100]. As a representative example, when hexagonal boron nitride (h-BN) nanosheets are aligned along a specific direction, inter-filler contact and percolation are more readily established along that direction, which markedly changes the internal thermal conduction pathways of the composite [101]. In magnetic-field-induced h-BN alignment systems, the dense and ordered arrangement can also lengthen the diffusion path of combustible pyrolysis products and suppress heat transfer into the interior, thereby improving thermal degradation and combustion behavior [102]. For 1D fillers such as CNTs, however, a high degree of alignment can reduce the number of effective interparticle contacts and thus decrease the connectivity of continuous percolated networks [73]. In PMMA/multi-walled carbon nanotube (MWCNT) composites, the disordered network maintains higher melt viscosity and forms a more coherent protective layer during combustion, whereas the oriented network evolves into a looser structure and shows weaker flame-retardant performance (Figure 7d) [103]. This change in network topology affects not only the shear-thinning and viscoelastic response of the polymer melt at elevated temperatures, but also the mechanical stability of the residual-char skeleton during combustion. Overall, the spatial orientation of nanofillers defines the geometric boundary conditions inside the composite and therefore provides the structural basis for tailoring direction-dependent physical barriers and fire-safety performance.

### 3.4. Multi-Mechanism Coupling and Hybrid Design

Whether multi-mechanism coupling gives rise to synergy depends mainly on whether the temporal sequence and phase distribution of different components can be properly coordinated during pyrolysis. In complex systems such as intumescent flame retardant (IFR) systems, acid-source decomposition, gas evolution, melt swelling, and char solidification must occur within mutually compatible time windows. If any of these processes occur too early or too late, the continuity of the resulting protective layer is compromised [104,105]. For example, in thermoplastic polyurethanes (TPU)/MAPP@Salen-Cu systems, rapid low-temperature charring must be followed by esterification- and crosslinking-driven expansion in the appropriate sequence to rapidly form a stable surface layer, thereby achieving dripping suppression and self-extinction [42].

In multiphase matrices, the selective localization of flame-retardant components and nanofillers can direct char formation to specific phase domains or interfacial regions. In immiscible linear low-density polyethylene (LLDPE)/polyamide 6 (PA6) blends, for example, when IFR components and nanosilicates or CNTs are enriched in different polymer phases, an intact and well-expanded char layer is more readily formed. By contrast, improper localization tends to produce a porous or discontinuous residual char [106,107]. In addition, the migration of nanosilica toward the surface during combustion leads to the accumulation of silicon-containing species at the char exterior, thereby improving the continuity of the outer-layer structure [108]. Beyond the formation site itself, nanofillers can also alter the development rate of the protective layer and its macroscopic and microscopic stability. In IFR systems containing halloysite nanotubes (HNTs), for instance, the nanofillers not only participate in char formation but also influence the development rate, microstructure, and mechanical stability of the protective layer, allowing the char layer to form more rapidly while maintaining good integrity [109]. Therefore, the final spatial distribution of the components and their participation in the same charring process further affect char morphology.

Multi-mechanism hybridization does not necessarily lead to synergy. When nanofillers such as graphene or excessive silica continuously increase the viscoelastic modulus of the polymer melt, the resulting high-viscosity melt raises the resistance to bubble nucleation and growth and thus suppresses foaming and volumetric expansion in IFR systems [35,108]. Under these conditions, excessive dispersion or network reinforcement can restrict melt flow and shift the multi-component interaction from synergy to antagonism [110]. Therefore, whether multi-mechanism synergy can be achieved depends on the sequence of component decomposition, phase localization, and whether melt rheology can be coordinated with the same charring process. Because the flame-retardant effectiveness of nanofillers depends strongly on polymer matrix, filler loading, dispersion state, surface chemistry, and testing conditions, direct numerical ranking across different studies may be misleading. A more appropriate comparison is to summarize the dominant action mechanisms, advantages, and limitations of representative nanofillers. Table 2 provides such a comparison for the main nanofiller types discussed in this review.

## 4. Impact of Nanomaterials on Polymer Fire Behavior

The structural factors and functional mechanisms discussed above are ultimately reflected in the macroscopic fire behavior of polymer nanocomposites. The roles of nanofillers during combustion further influence key fire characteristics, including ignition, thermal degradation, heat release, flame spread, and smoke emission. Building on the preceding discussion of functional mechanisms and regulatory factors, this section examines the effects of nanofillers on polymer fire safety in terms of time to ignition, thermal stability, heat release rate (HRR), flame spread behavior, and smoke emission and toxicity, and further establishes the relationships among structure, mechanism, and performance.

### 4.1. Time to Ignition

**Figure 8:**
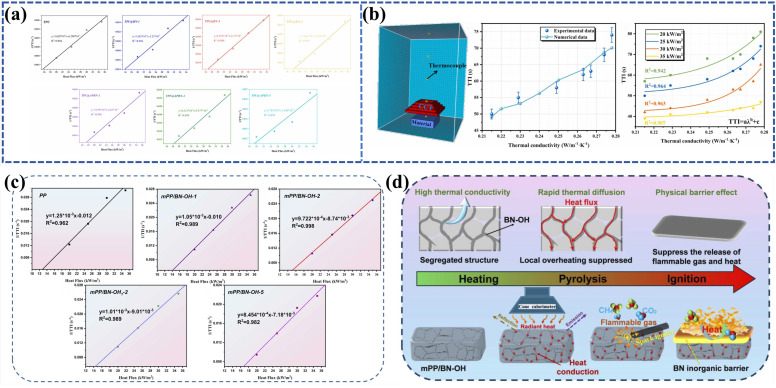
(**a**,**b**) Linear fitting of 1/TTI versus external heat flux and thermal-diffusion-based analyses of the relationship between thermal conductivity and TTI in TPU and its h-BN/APBN composites. Reproduced with permission from ref. [112]. (**c**,**d**) Linear fitting of 1/TTI versus external heat flux and schematic delayed-ignition mechanism in PP composites with a segregated BN-OH structure. Reproduced with permission from ref. [118].

Time to ignition (TTI) is the time required for a material to reach sustained flaming under a specified incident heat flux. In fire safety assessment, TTI mainly reflects how readily a material ignites and usually needs to be evaluated alongside heat release and related fire behaviors [70]. From the perspective of ignition analysis, the relationship between TTI and external heat flux can be described using thermally thin or thermally thick assumptions, with the goodness of fit indicating the governing ignition regime [14]. The pre-ignition surface temperature rise, pyrolysis kinetics, and the release rate of combustible volatiles are the main factors controlling TTI [4]. In polymer nanocomposites, nanofillers alter the heat transfer and pyrolysis processes of the matrix before ignition, so variations in TTI are often strongly mechanism-dependent [111]. Zhang et al. further show that TTI is closely related to thermal conductivity, and that this relationship can be examined by combining linear fitting, experimental results, and thermal-diffusion-based analysis, as shown in Figure 8a,b [112].

Under certain conditions, some nanofillers can shorten TTI and induce premature ignition of the matrix. At the physical level, this is usually associated with increased absorption of radiant heat at the material surface. For example, the addition of CNTs increases the surface absorptivity of polypropylene (PP) to thermal radiation, accelerates surface heating, and causes the specimen to reach its ignition temperature more rapidly [113]. At the chemical level, early catalytic degradation caused by fillers can also shorten TTI. The early decomposition of BP causes the system to enter the pyrolysis stage earlier, and the premature release of combustible volatiles leads to a marked reduction in TTI [54]. In addition, filler interfacial characteristics also affect early volatile release. Conventional ammonium-modified nanoclay promotes the early degradation of PA6, leading to a significantly shortened TTI, whereas shielding this interfacial activity with a polymer coating restores the TTI of the composite to a level close to that of the neat resin [114].

By contrast, when nanofillers provide endothermic buffering or form a surface barrier before ignition, TTI is usually prolonged. In polylactic acid (PLA) systems, trace amounts of zeolitic imidazolate framework-67 (ZIF-67) restrict initial heat and mass transfer via their porous architecture, and the pre-ignition endothermic decomposition of ZIF-67 consumes part of the incident heat, thereby delaying ignition [115]. In EP, a CoAl-LDH@ZIF-67 hybrid structure can also delay ignition by acting as a heat-flow barrier before flaming begins [116]. Compared with bulk blending, localized distribution of nanofillers at the material surface often plays a more pronounced role in extending TTI. After constructing LDH nanocomposite coatings on the surfaces of ethylene-vinyl acetate copolymer (EVA) or ethylene-butyl acrylate copolymer (EBA), the surface enrichment of 2D nanosheets slows early heat transfer and volatile escape, effectively prolonging ignition time [117]. Similarly, after constructing a segregated BN-OH structure in PP, 5 wt% BN-OH increases thermal conductivity by 42.4% and increases TTI by 35.3% under 35 kW/m^2^. This effect arises not only from rapid heat dissipation, but also from the continuous BN-OH barrier suppressing the diffusion of pyrolysis products [118]. The linear fitting relationship between 1/TTI and external heat flux and the corresponding delayed-ignition mechanism are shown in Figure 8c and Figure 8d, respectively.

### 4.2. Thermal Stability

The thermal stability of polymer nanocomposites is usually evaluated by a combination of parameters, including the temperature at 5% mass loss (T_5%_), the temperature at the maximum mass loss rate (T_max_), and the char residue at high temperature. The influence of nanofillers on the thermal degradation of the matrix does not always manifest as an overall increase in characteristic degradation temperatures, but depends strongly on the physical barrier network formed by the fillers and on their chemical reactivity and catalytic activity. Therefore, the thermal stability of such materials should not be judged solely from the change in a single characteristic degradation temperature, but should be assessed alongside the entire degradation profile and the charring behavior at high temperatures [119,120,121].

**Figure 9:**
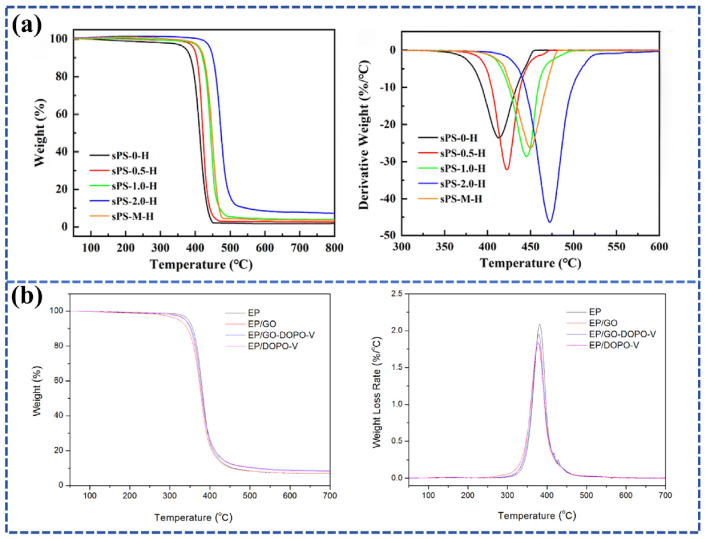
(**a**) TGA/DTG curves showing the effects of D-MXene on the thermal degradation behavior of sPS. Reproduced with permission from ref. [122]. (**b**) TGA/DTG curves showing the effects of GO-DOPO-V on the thermal degradation behavior of EP. Reproduced with permission from ref. [123].

For systems dominated by lamellar barrier effects, improved thermal stability usually manifests as an overall increase in the characteristic degradation temperatures. In modified MXene/syndiotactic polystyrene (sPS) nanocomposites, for example, 2.0 wt% D-MXene increases the T_5%_ and T_max_ of the matrix by 71.2 °C and 59.2 °C, respectively, while the char residue at 800 °C increases from 1.72 wt% to 7.85 wt% (Figure 9a) [122]. This result indicates that when 2D nanofillers form a highly dispersed physical barrier and effectively lengthen the diffusion path of volatile pyrolysis products, the stability of the material can be improved consistently across the entire thermal decomposition process.

Unlike the overall retardation produced by purely physical barriers, systems containing reactive or catalytic components often show slightly earlier initial decomposition but improved char retention at high temperatures. For example, in the GO-DOPO-V/EP system, the reactive components decrease the T_5%_ of the matrix from 346.0 °C to 338.4 °C, whereas the final char residue increases from 7.02 wt% to 8.52 wt% (Figure 9b) [123]. Similarly, in the EP system containing iron-loaded polydopamine-modified MMT, the catalytic charring effect lowers the T_5%_ of the matrix slightly from 370 °C to 367 °C, while the char residue at 600 °C increases markedly from 16 wt% to 24 wt% [124]. A common feature of these systems is that they alter the degradation and crosslinking pathways of the matrix to accelerate the formation of a compact char layer, thereby providing better thermal protection at elevated temperatures.

### 4.3. Heat Release Rate

HRR and its derived parameters, including peak heat release rate (PHRR), total heat release (THR), and maximum average rate of heat emission (MARHE), are key metrics for evaluating the fire hazard of polymers. PHRR characterizes the maximum intensity of heat release during combustion, whereas THR reflects the total heat released over the entire burning process [125]. MARHE represents the maximum value of the averaged heat release rate over time and is often used to evaluate the overall fire growth and heat-release hazard of a material. A lower MARHE value generally indicates a reduced heat-release intensity and, therefore, improved fire safety. For polymer nanocomposites, changes in heat-release behavior usually first appear as a pronounced decrease in PHRR, while the change in THR is relatively limited [105,126]. In many nanofiller systems dominated by physical barriers, when the effective heat of combustion remains nearly unchanged, the reduction in HRR mainly corresponds to a lower mass loss rate in the condensed phase. In other words, nanofillers primarily reduce the flux of combustible volatiles released per unit time, but do not necessarily substantially decrease the total fuel mass that ultimately participates in combustion.

**Figure 10:**
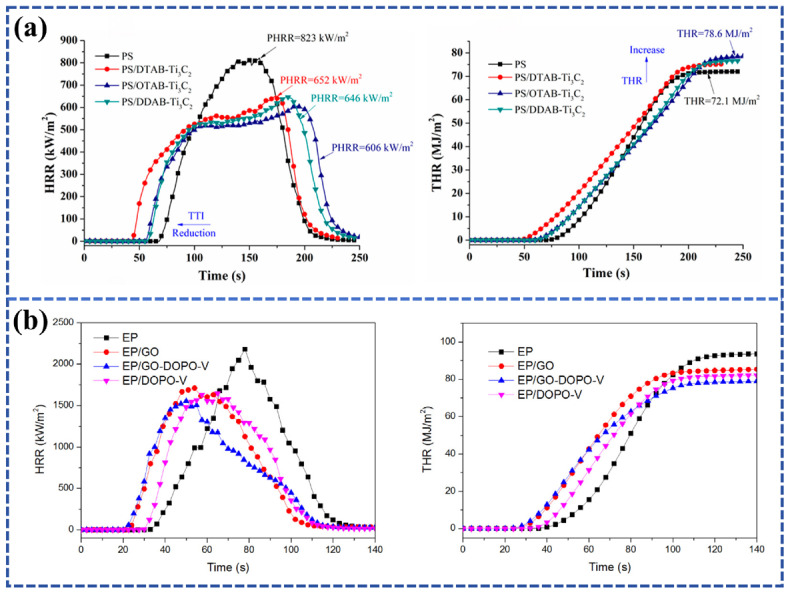
(**a**) HRR and THR curves of modified MXene/PS systems. Reproduced with permission from ref. [90]. (**b**) HRR and THR curves of GO-DOPO-V/EP systems. Reproduced with permission from ref. [123].

This feature is most typical of single-nanofiller systems dominated by physical barriers. In PA6 containing 5 wt% layered silicate, the PHRR decreases from 1011 kW/m^2^ to 361 kW/m^2^, corresponding to a reduction of about 63%, whereas the THR changes only slightly [31]. A similar trend is observed in PMMA/SWNT systems: 0.5 wt% of well-dispersed SWNTs lowers the PHRR by more than 50%, while the THR of the samples remains essentially comparable to that of the neat matrix [20]. Likewise, in modified MXene/PS systems, although the PHRR decreases from 823 kW/m^2^ to 606 kW/m^2^, the THR instead increases from 72.1 MJ/m^2^ to 78.6 MJ/m^2^ (Figure 10a) [90]. These results indicate that the more common role of a single nanofiller is to suppress the heat-release peak and prolong the burning process, rather than to reduce the total heat released.

When a nanosystem combines physical barrier effects with chemical intervention, PHRR and THR may decrease simultaneously. In a PP system containing 5 wt% PMGO and 20 wt% IFR, the PHRR and THR decrease by 61.5% and 40.2%, respectively [127]. In GO-DOPO-V/EP, a 2 wt% loading reduces both parameters by 28.8% and 15.6%, respectively (Figure 10b) [123]. Likewise, BP/CNT nanohybrids in EP decrease the PHRR and THR by 55.81% and 41.17%, respectively [128]. Compared with single-nanofiller systems, the role of these synergistic systems is no longer limited to suppressing the heat-release peak, but extends to reducing the total heat ultimately released during combustion.

### 4.4. Flame Spread Behavior

Flame spread reflects the ability of the flame front to continuously propagate into unburned regions [6]. Controlling flame spread should not rely solely on improving a single static combustion metric, but on whether the heat feedback from the flame front is sufficient to sustain continuous pyrolysis in the unburned region ahead of the front [129]. In thermoplastic polymers, melting, flow, and dripping during combustion redistribute surface fuel and alter the original heat-transfer pathways [59,130]. As a result, the flame spread rate (FSR) often shows no direct correspondence with conventional metrics such as limiting oxygen index (LOI), TTI, or PHRR [131]. During the early stage of surface flame propagation, the coupling among heat feedback from the flame front, phase change, and melt migration often determines the subsequent growth of the fire [132]. Understanding this combustion behavior dominated by continuous surface propagation is particularly important when assessing the fire hazards of thermoplastic interior materials, flexible foams, and other components prone to surface flame spread [133].

**Figure 11:**
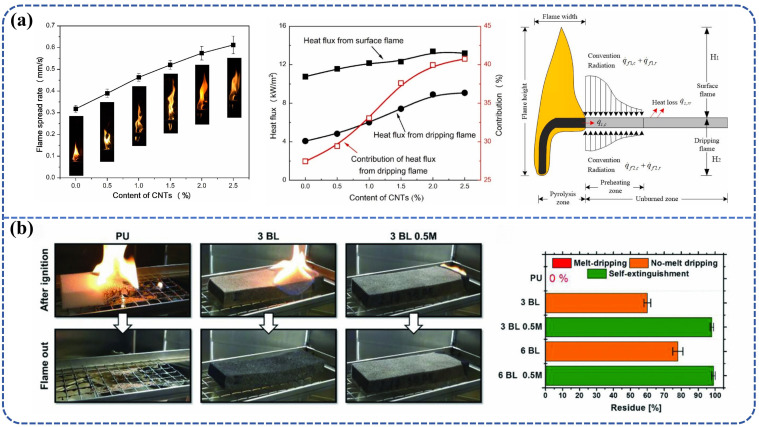
(**a**) Effects of CNT loading on flame spread rate, heat-feedback contribution from the dripping flame, and the corresponding simplified heat-transfer model during flame spread over PMMA composites. Reproduced with permission from ref. [134]. (**b**) Suppression of surface flame spread and promotion of self-extinction in flexible PU foams by a GO exoskeleton. Reproduced with permission from ref. [136].

The influence of nanofillers on flame spread should also be interpreted within this dynamic heat-transfer framework [129,130]. In bulk-filled systems, improvements in static combustion metrics do not always translate into suppression of dynamic flame spread. In PMMA/MWCNT composites, for example, the addition of MWCNTs increases the LOI from 18% to 20% and decreases the PHRR from 548 kW/m^2^ to 372 kW/m^2^, yet the FSR increases from 0.28 mm/s to 1.1 mm/s [131]. At an MWCNT loading of 2.5 wt%, the heat flux supplied by the dripping flame is about twice that of neat PMMA and accounts for 40% of the total heat flux at the flame front [134]. This indicates that the acceleration of flame spread arises mainly from the strong enhancement of frontal heat feedback by flaming drips, rather than from a deterioration in the overall static flame-retardant performance of the material. The corresponding increase in flame spread rate, the enhanced contribution of the dripping flame to frontal heat feedback, and the associated heat-transfer model are shown in Figure 11a [134].

Compared with the limited ability of bulk filling to control melt migration, interfacial nanostructuring can more directly interrupt flame propagation in porous materials dominated by surface burning, such as PU foams [133]. Studies have shown that CNT coatings and GO exoskeletons can effectively block flame spread on the surface of flexible PU foams [135,136]. In particular, GO-exoskeleton-treated samples can transition from sustained burning to self-extinction, as shown by the corresponding combustion processes and residue states in Figure 11b. Overall, improving the flame-spread resistance of nanocomposites does not depend on simply lowering a specific combustion parameter, but on whether the nanostructure can effectively weaken frontal heat feedback and suppress the continuous advance of the surface flame. This is the main reason why surface nanostructures are of particular safety significance for controlling fire spread in materials.

### 4.5. Smoke Emission and Toxicity

In polymer fires, the risks of incapacitation and death arise mainly from combined exposure to toxic gases such as CO and HCN and to smoke particles. An increase in the fractional effective dose (FED) generally indicates a greater risk of fire-induced injury and death [137]. As a fire shifts from well-ventilated to under-ventilated conditions, complete combustion is suppressed and the products of incomplete combustion increase markedly. As a result, the yields of CO and HCN rise, and fire toxicity becomes significantly more severe [10,138]. For this reason, relying solely on gas-phase radical scavenging to weaken flame chain reactions does not guarantee a simultaneous reduction in overall fire hazard. If condensed-phase charring and subsequent oxidation are not strengthened at the same time, the proportion of toxic gases in the fire effluents may still increase even when heat release decreases [139].

The effect of nanofillers on smoke emission is first reflected in their physical regulation of pyrolysis product transport. Once high-aspect-ratio 2D components form percolated networks or continuous barrier layers during heating, vigorous bubbling within the melt is suppressed, the escape of volatile pyrolysis products and condensable aromatic intermediates is inhibited, and mass exchange between the gas and condensed phases slows accordingly. As a result, the residence time of pyrolysis fragments in the condensed phase is prolonged, making secondary cross-linking, aromatization, and char densification more likely and thereby reducing both the formation and release of smoke particles [22,140]. This pathway has been verified in various 2D systems. For example, in graphene/BP hybrid systems, both the smoke production rate (SPR) and the CO release rate decrease markedly [141]. Similarly, in MXene/chitosan surface nanostructured systems, total smoke production, peak smoke production rate, and CO release all decrease simultaneously [142].

Physical barriers alone are still insufficient to fully control the increase in toxicity caused by incomplete combustion, whereas the introduction of transition-metal nodes further endows the system with interfacial catalytic activity [143,144]. Hybrid structures containing Co, Ni, Fe, or Cu, such as LDHs, MOF-derived oxides, and metal oxides, can form interfacial sites with both Lewis acidity and redox activity at elevated temperatures. On the one hand, these sites promote dehydration cross-linking, aromatization, and charring, thereby improving the integrity and stability of the residual char layer. On the other hand, they accelerate the further oxidation of incomplete-combustion intermediates such as CO at the gas–solid interface, thereby reducing both smoke emission and toxic gas release [145]. In MOF-derived double-shelled metal oxide nanocage/EP systems, for example, the peak smoke production rate (PSPR), total smoke production (TSP), and CO release are all reduced [146]. Overall, nanocomposite systems that combine continuous barrier layers with interfacial catalytic sites are more likely to improve smoke behavior, reduce CO emissions, and mitigate fire toxicity simultaneously.

## 5. Conclusions and Perspectives

This review has systematically summarized the roles of nanofillers in the fire safety of polymer nanocomposites across three interconnected levels: functional mechanisms, regulatory factors, and macroscopic fire behavior. Existing studies show that the fire-safety effects of nanofillers do not arise from any single flame-retardant action. Instead, nanofillers simultaneously influence heat transfer, mass transfer, free-radical reactions, and high-temperature rheological behavior in both the condensed and gas phases, thereby altering the entire combustion process from thermal degradation and ignition to heat release, flame spread, and smoke and toxic-gas emission. Physical barriers delay the exchange of heat, oxygen, and combustible volatiles; catalytic charring improves carbon retention and char stability in the condensed phase; radical scavenging weakens chain reactions in the flame zone; and viscoelastic networks further affect bubbling, dripping, and char formation. Together, these micro- and mesoscale effects are ultimately reflected in systematic changes in ignitability, thermal stability, heat release, flame spread, and smoke toxicity.

Furthermore, the ultimate fire-safety benefits of polymer nanocomposites are not determined solely by filler type, but by whether an effective match can be achieved among structural state, action sequence, and combustion scenario. The geometric dimensions and loading levels of fillers determine whether percolated networks and active-site densities can reach an effective operating range. Interfacial compatibility and dispersion state determine the continuity of barrier pathways and the accessibility of chemical sites. Spatial orientation and phase localization further affect the direction of heat and mass transfer, the location of char formation, and melt-flow behavior. It should also be noted that multi-mechanism hybridization does not automatically lead to synergy. Only when acid-source decomposition, matrix degradation, melt thickening, gas evolution, and char solidification occur within mutually compatible temperature–time windows can different mechanisms form a continuous response during the same combustion process. Therefore, the key to flame-retardant nanocomposite design is no longer the simple addition of functional components, but rather the effective matching of different structural units with the combustion process in time and space.

Despite the substantial progress made in this field, several major limitations remain. Fire-safety evaluation still relies heavily on static metrics such as LOI, UL-94, and PHRR, while large differences in specimen thickness, heat flux, geometry, and ventilation conditions across studies weaken the scientific basis for absolute numerical comparisons. In addition, current mechanistic understanding still relies mainly on ex situ characterization, and direct in situ evidence remains insufficient for clarifying the transient coupling among char growth, bubble evolution, interfacial mass transfer, network reconstruction, high-temperature rheological instability, and frontal heat feedback during real combustion. Furthermore, high efficiency at low loading under laboratory conditions does not necessarily ensure stable performance in engineering applications. For nanoscale flame-retardant systems, filler dispersion, interfacial stability, and structural retention at high loading levels, together with environmental and health safety during long-term service, remain key constraints on practical application.

Future research should focus on transforming scattered performance observations into verifiable structure–mechanism–property relationships under unified boundary conditions. At the evaluation level, this requires the gradual establishment of comprehensive criteria covering ignition, heat release, dripping, flame spread, and smoke toxicity. At the mechanistic level, greater emphasis should be placed on in situ identification of key processes such as nanoscale network migration, char evolution, interfacial catalysis, and high-temperature rheological instability. On this basis, structural parameters such as filler decomposition temperature range, catalytic activity window, phase distribution, and rheological percolation threshold should be incorporated into a unified design framework. As evaluation boundaries become clearer and mechanistic evidence continues to accumulate, data-driven methods, including machine learning, can serve as useful tools for multi-objective performance prediction and candidate system identification. In particular, descriptors such as polymer matrix, nanofiller type, loading level, surface chemistry, dispersion state, processing conditions, and testing parameters could be linked with fire-safety outputs such as TTI, PHRR, THR, LOI, char yield, smoke production, and toxic-gas release. However, such approaches still require standardized datasets, comparable testing conditions, and mechanism-informed feature selection to avoid purely empirical correlations. At the same time, future design should incorporate sustainability-related criteria, including low toxicity, halogen-free composition, smoke/toxicity reduction, recyclability, reprocessability, and life-cycle considerations, into the fire-safety evaluation framework. Only in this way can fire-safety research on polymer nanocomposites move beyond empirical performance optimization and toward rational, sustainable, and application-oriented design.

## Figures and Tables

**Table 1:** Representative acid-source and solid-acid-related systems involved in catalytic charring.

System	Acidic Species or Active Sites	Supported Role in Catalytic Charring	Refs.
Polyphosphate/APP-based systems	Phosphoric or polyphosphoric acid species generated during thermal decomposition	Provide acid sources that catalyze dehydration and cross-linking reactions, promoting char formation in intumescent systems.	[13,37]
α-ZrP-based intumescent systems	Proton acid sites and Lewis acid sites associated with α-zirconium phosphate	Catalyze carbonization/dehydrogenation and promote carbonaceous char formation in intumescent PP systems.	[41]
Phytic-acid-functionalized UiO-66-NH_2_	Phosphate-rich groups introduced by phytic acid on UiO-66-NH_2_	Regulates epoxy pyrolysis and promotes compact, cross-linked char formation.	[36]

**Table 2:** Comparison of main mechanisms, advantages, and limitations of representative nanofillers.

Nanofiller Type	Main Action Mechanism	Advantage	Disadvantage
Layered silicates/MMT	Intercalated or exfoliated layers create tortuous paths and may accumulate in the surface char, slowing heat, oxygen, and volatile transport.	Representative barrier-type nanofillers; useful for lowering heat-release rate when dispersion and exfoliation are sufficient.	Effectiveness depends on polymer–clay interfacial chemistry and dispersion; some organoclay systems may promote earlier degradation or shorten TTI.
LDHs	Layered barrier effect, endothermic decomposition, gas dilution, and formation of metal-containing residues that can support char formation.	Composition and interlayer chemistry are tunable; suitable for hybrid fillers and coating-type designs.	Performance is formulation-dependent; relatively high loading or poor dispersion can limit processing and mechanical benefits.
CNTs	Formation of a percolated network that modifies high-temperature rheology, improves melt strength, and stabilizes residue structures.	Can reduce PHRR at low loading when well dispersed; particularly relevant to melt-rheology and dripping control.	Dispersion is difficult; improved static cone-calorimeter metrics do not necessarily imply reduced flame spread in dripping-prone systems.
Graphene/GO	Two-dimensional barrier effect, char reinforcement, and functionalized interfaces for coupling with phosphorus-, nitrogen-, silicon-, or metal-containing species.	Strong platform for barrier-enhanced and chemically functionalized hybrid flame-retardant systems.	Restacking and poor compatibility reduce efficiency; excessive network reinforcement may interfere with intumescent expansion.
MXenes	Two-dimensional barrier effect combined with surface-termination-mediated interfacial regulation and transition-metal-containing condensed-phase action.	Surface chemistry enables modification and hybridization; useful for coupling barrier effects with char/smoke regulation.	Oxidation stability, restacking, and surface chemistry must be controlled to maintain performance.
MOFs/MOF derivatives	Metal nodes or derived metal oxides catalyze charring and can promote smoke/toxic-gas suppression in suitable systems.	Highly tunable metal nodes, ligands, and derivative structures; useful for catalytic char formation and smoke suppression.	Thermal stability, synthesis complexity, and matrix compatibility can limit direct generalization across polymers.
BP	Phosphorus-containing radical intervention combined with condensed-phase charring and nanosheet barrier effects.	Can provide both gas-phase and condensed-phase flame-retardant contributions, especially in protected or hybrid systems.	Sensitive to oxygen and moisture; premature decomposition may affect ignition behavior if not stabilized.
C_60_/DOPO-functionalized C_60_	C_60_ can trap macromolecular radicals; DOPO functionalization introduces phosphorus-containing radical-quenching activity.	Useful for discussing radical-intervention nanofillers beyond conventional barrier-dominated mechanisms.	Evidence is system-specific and usually requires good dispersion and complementary condensed-phase protection.
Hybrid nanofillers	Couple barrier effects, catalytic charring, radical intervention, and/or rheological regulation.	Suitable for multi-mechanism design and multi-objective fire-safety optimization.	Synergy is not automatic; mismatched decomposition windows, phase localization, or melt rheology may cause antagonistic effects.

## Data Availability

No new data were created or analyzed in this study. Data sharing is not applicable to this article.

## Abbreviations

The following abbreviations are used in this manuscript:

1D	one-dimensional
2D	two-dimensional
ATH	aluminum trihydroxide
BP	black phosphorus
CNT	carbon nanotube
DOPO	9,10-dihydro-9-oxa-10-phosphaphenanthrene-10-oxide
DTG	derivative thermogravimetry
EBA	ethylene-butyl acrylate copolymer
EP	epoxy resin
EVA	ethylene-vinyl acetate copolymer
FED	fractional effective dose
FSR	flame spread rate
GO	graphene oxide
h-BN	hexagonal boron nitride
HNT	halloysite nanotube
HRR	heat release rate
IFR	intumescent flame retardant
LDH	layered double hydroxide
LLDPE	linear low-density polyethylene
LOI	limiting oxygen index
MARHE	maximum average rate of heat emission
MMT	montmorillonite
MOF	metal–organic framework
MWCNT	multi-walled carbon nanotube
PA6	polyamide 6
PC	polycarbonate
PE	polyethylene
PHRR	peak heat release rate
PLA	polylactic acid
PMMA	poly(methyl methacrylate)
PP	polypropylene
PS	polystyrene
PSPR	peak smoke production rate
PU	polyurethane
RGO	reduced graphene oxide
SPR	smoke production rate
sPS	syndiotactic polystyrene
SWNT	single-walled carbon nanotube
TGA	thermogravimetric analysis
THR	total heat release
TPU	thermoplastic polyurethanes
TSP	total smoke production
TSR	total smoke release
TTI	time to ignition
UL-94	Underwriters Laboratories 94
ZIF-67	zeolitic imidazolate framework-67
